# Supplementary material for: Learning chemical sensitivity reveals mechanisms of cellular response
Source: Commun Biol. 2024 Sep 15;7:1149. doi: 10.1038/s42003-024-06865-4 (PMC11402971; doi:10.1038/s42003-024-06865-4)
Supplement: Supplementary file 2 — Supplementary Information [file 42003_2024_6865_MOESM2_ESM.pdf]

Supplementary Information for
Learning chemical sensitivity reveals mechanisms of cellular response

William Connell[1,2,3], Kristle Garcia[3,4,5,6], Hani Goodarzi[3,4,5,6], Michael J. Keiser[1,2,3]

1. Department of Pharmaceutical Chemistry, University of California, San Francisco, San Francisco, CA, USA
2. Institute for Neurodegenerative Diseases, University of California, San Francisco, San Francisco, CA, USA
3. Bakar Computational Health Sciences Institute, University of California, San Francisco, San Francisco, CA, USA
4. Department of Biochemistry and Biophysics, University of California, San Francisco, San Francisco, CA, USA
5. Department of Urology, University of California, San Francisco, San Francisco, CA, USA
6. Helen Diller Family Comprehensive Cancer Center, University of California, San Francisco, San Francisco, CA, USA

Correspondence: keiser@keiserlab.org

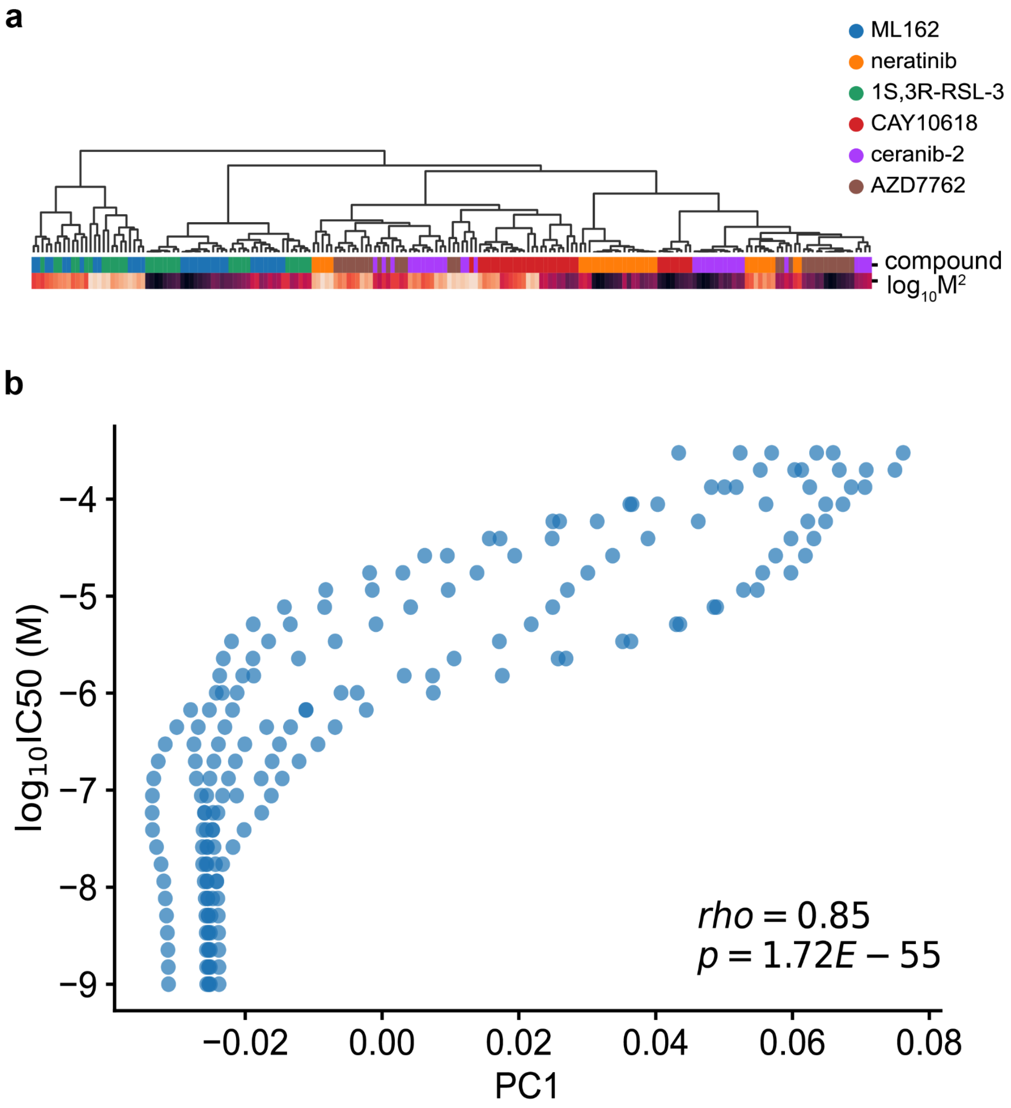

Supplementary Figure 1 | Conditional parameter analysis.

(a) Hierarchical clustering of learned gamma parameters. Color bars indicated compound identity and squared compound concentration. (b) Relationship between principal component 1 (PC1) of learned beta parameters and input concentration; Pearson correlation coefficient.

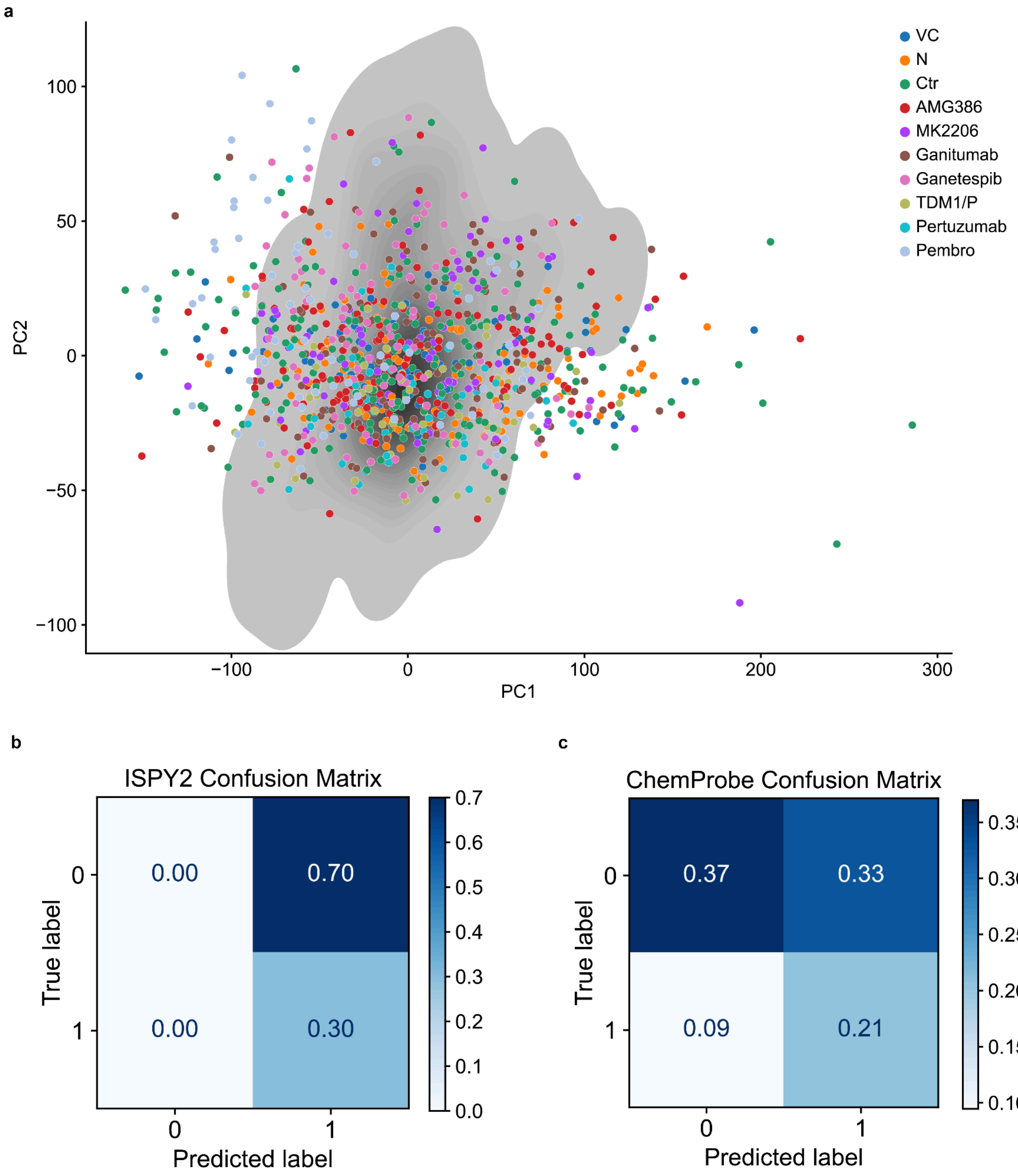

Supplementary Figure 2 | I-SPY2 analysis.

(a) I-SPY2 participant tumor gene expression profiles projected on the CCLE training data distribution. VC, veliparib/carboplatin; N, neratinib; Ctr, control; gray = kernel density estimate (KDE) of training data distribution. (b) I-SPY2 confusion matrix of predicted response and observed response. (c) ChemProbe confusion matrix of predicted response and observed response.

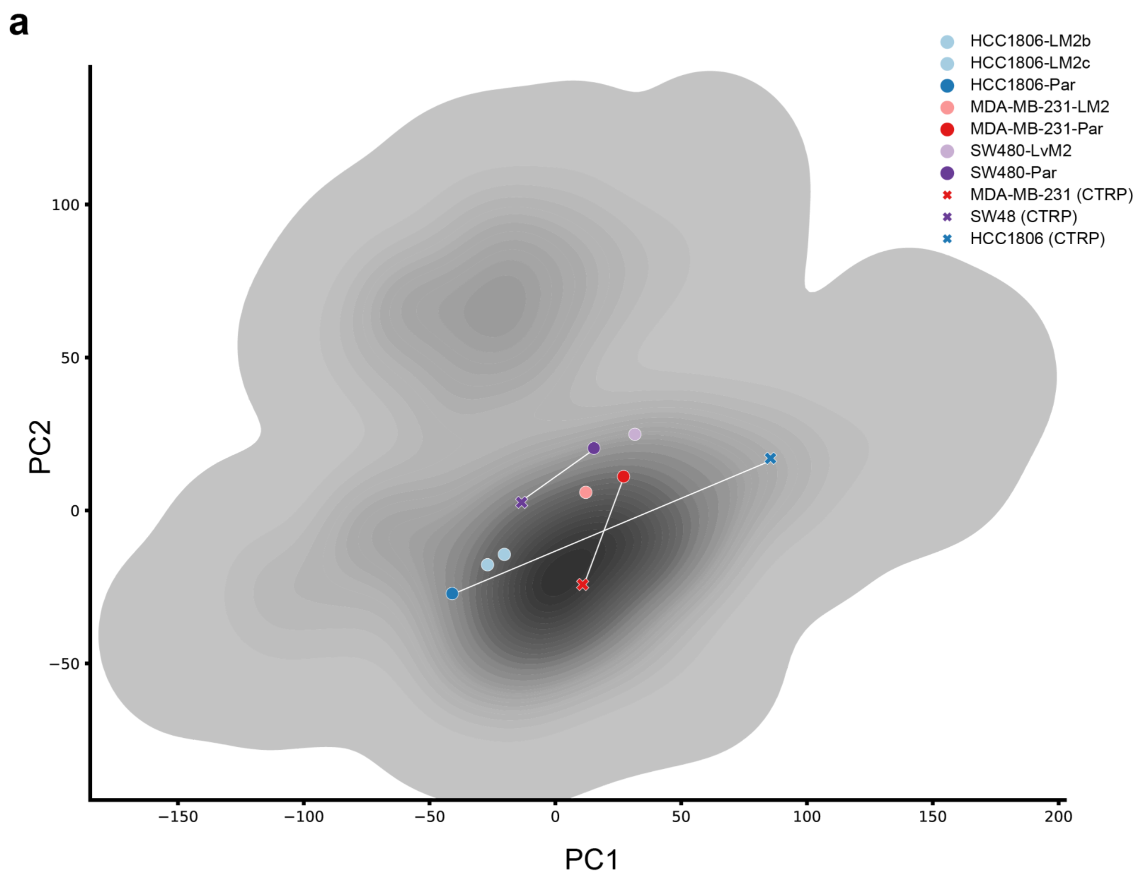

Supplementary Figure 3 | Prospective cell line expression similarity analysis.

(a) PCA of gene expression similarity between prospectively tested cell lines and matched counterparts in the CTRP training data. gray = KDE of training data distribution.

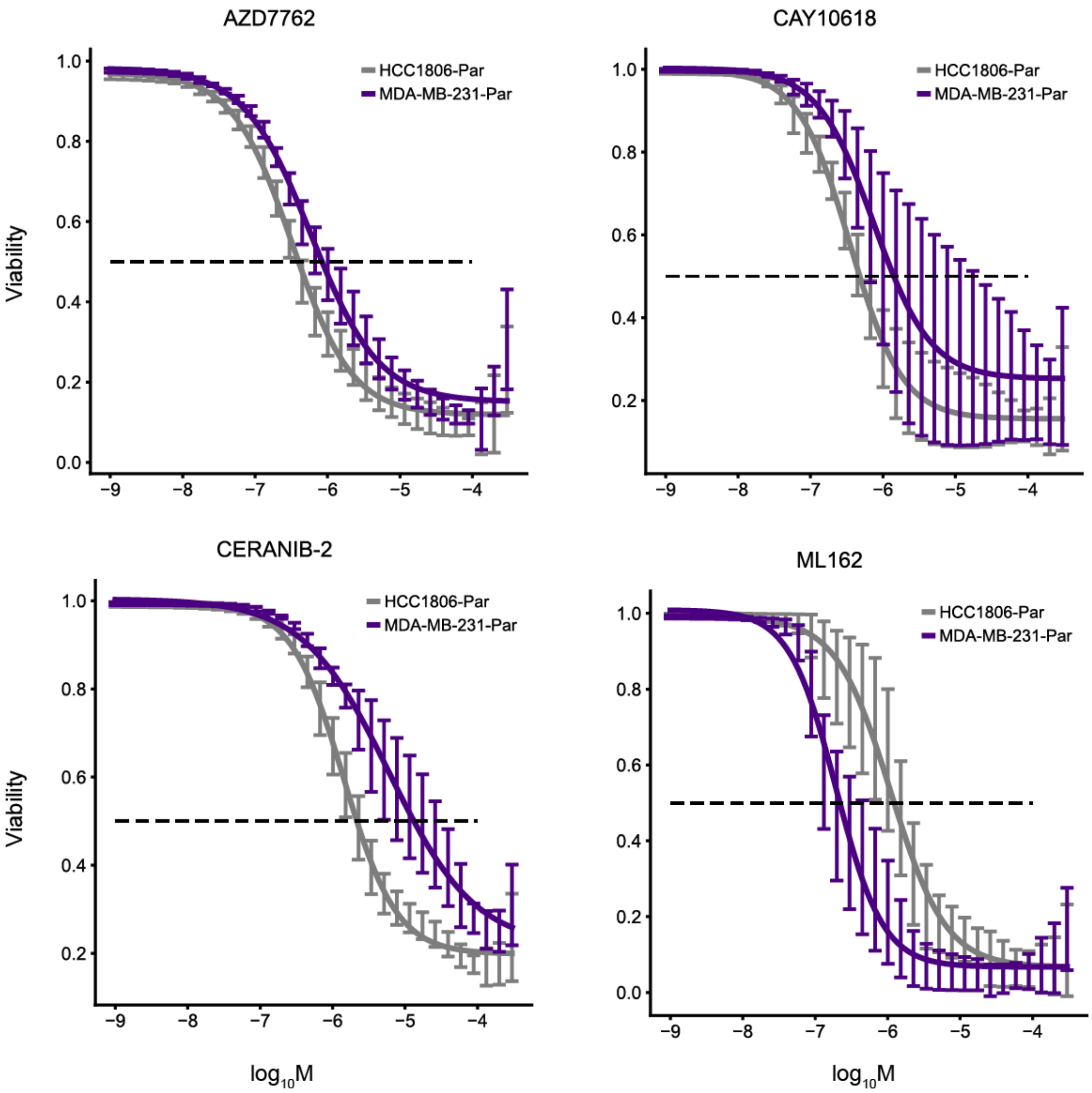

Supplementary Figure 4 | *In silico* dose-response curves.

Predicted dose-response relationships of HCC1806 and MDAMB231 response to AZD7762 (n=5 independent samples), CAY10618 (n=5 independent samples), ceranib-2 (n=5 independent samples), and ML162 (n=5 independent samples). 95% confidence intervals.

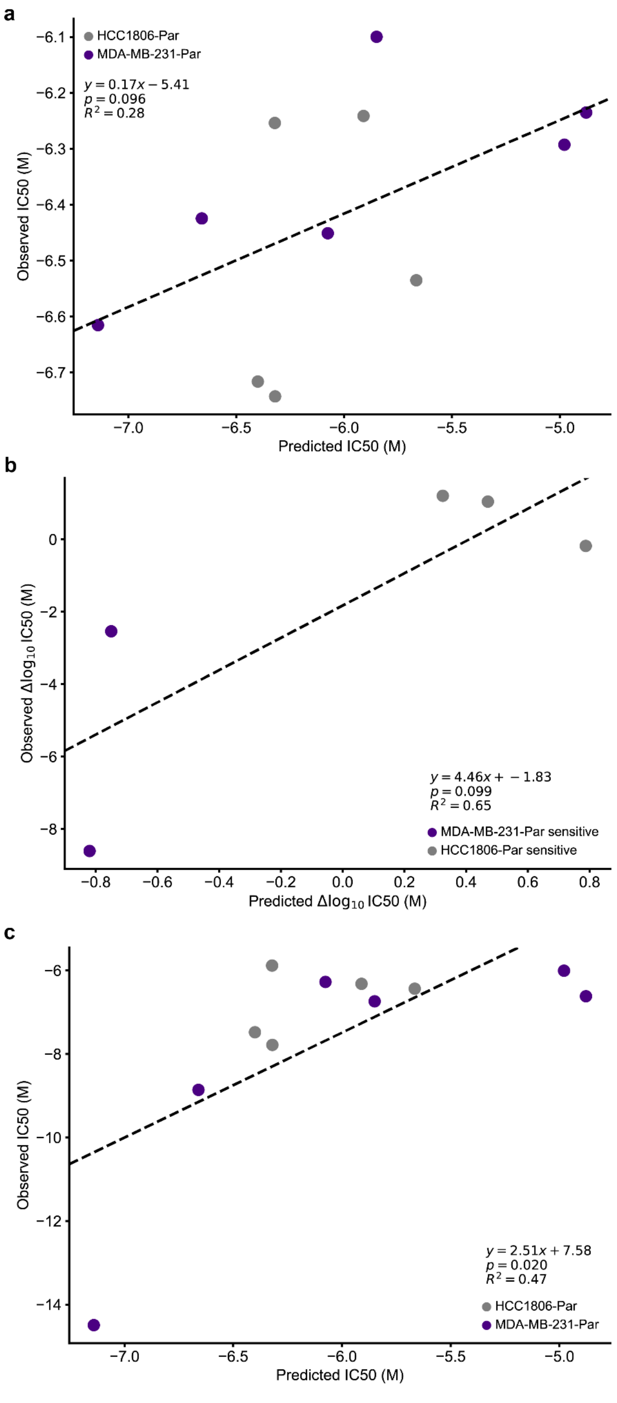

Supplementary Figure 5 | Prospective dose-response analysis.

(a) Relationship between predicted and observed IC50s of prospectively tested compounds in HCC1806-Par and MDA-MB-231-Par cell lines. Two-sided t-test (n=11 independent experiments). (b) Calibration experiment; relationship between predicted difference in IC50 and

observed difference in IC50 between HCC1806-Par and MDA-MB-213-Par across tested compounds. Two-sided t-test (n=5 independent experiments). (c) Calibration experiment; relationship between predicted and observed IC50s of prospectively tested compounds in HCC1806-Par and MDA-MB-231-Par cell lines. Two-sided t-test (n=11 independent experiments).

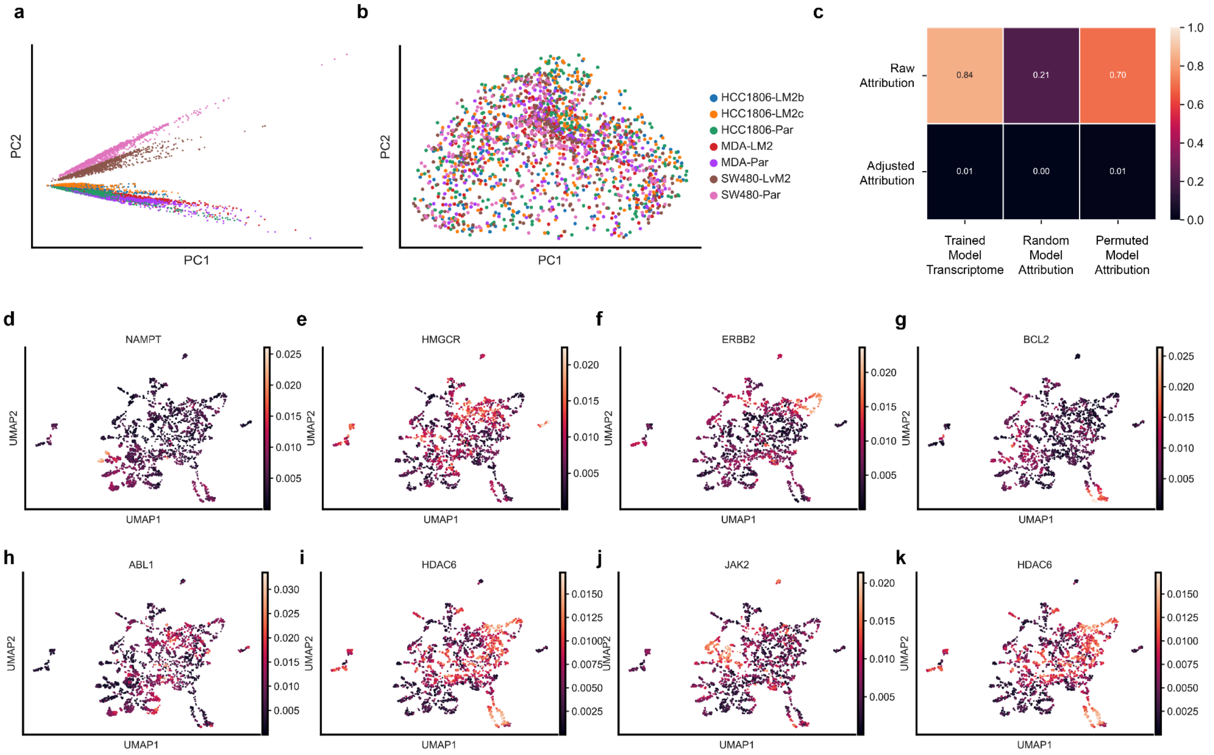

Supplementary Figure 6 | Attribution analysis.

PCA decompositions of (a) raw attribution vectors versus (b) adjusted attribution vectors. (c) Pearson correlation between raw attributions and adjusted attributions versus transcriptome inputs, randomly initialized model attributions, and attributions of a model trained on permuted labels. (d-g) UMAP of cell line-compound attribution samples colored by compound targets with specific attributions. (h-k) UMAP of cell line-compound attribution samples colored by compound targets with diffuse attributions.

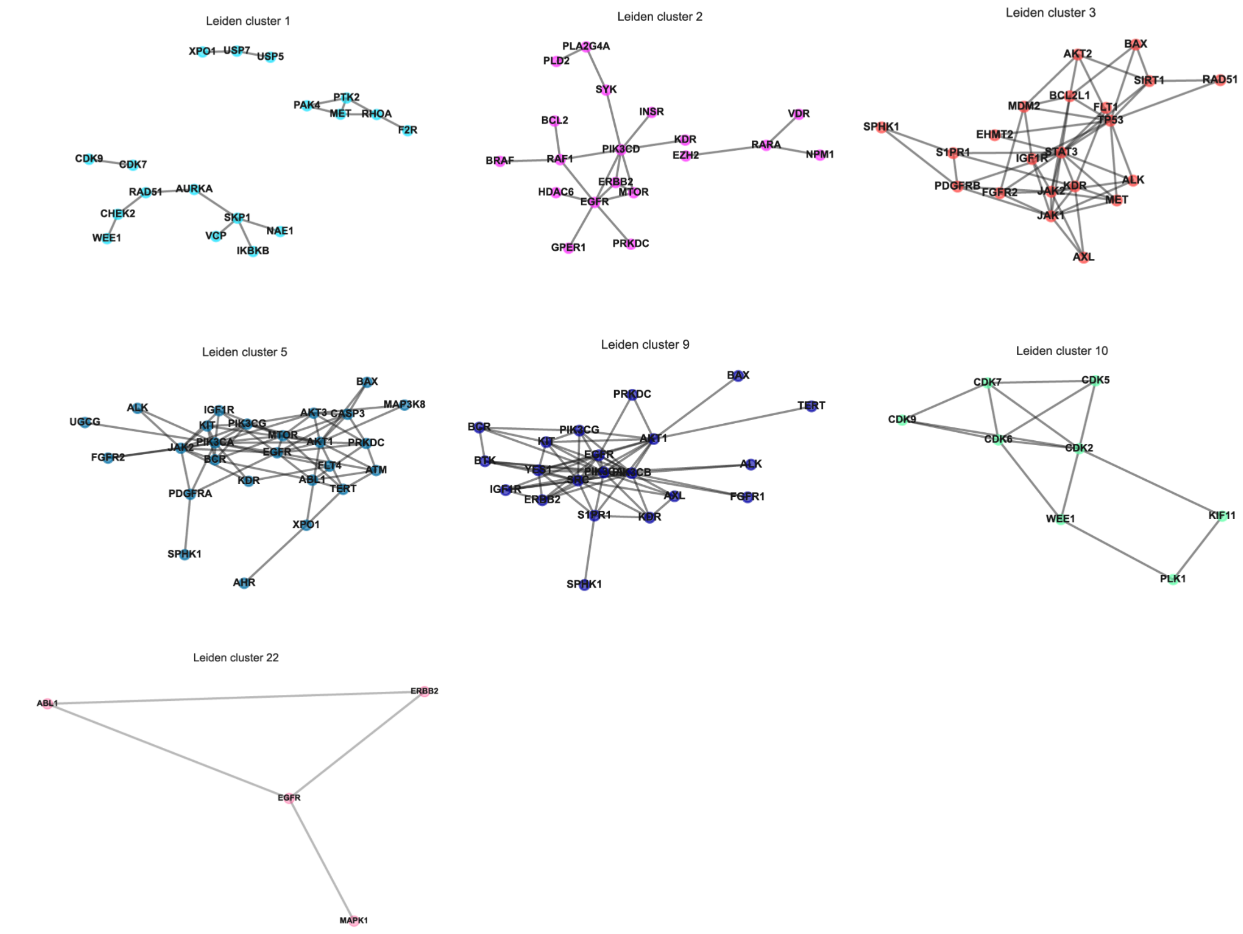

Supplementary Figure 7 | Module of action (ModOA) clustered target subgraphs.

Network representation of ModOA with significant enrichment of protein-protein interactions. Node color reflects Leiden cluster assignment.

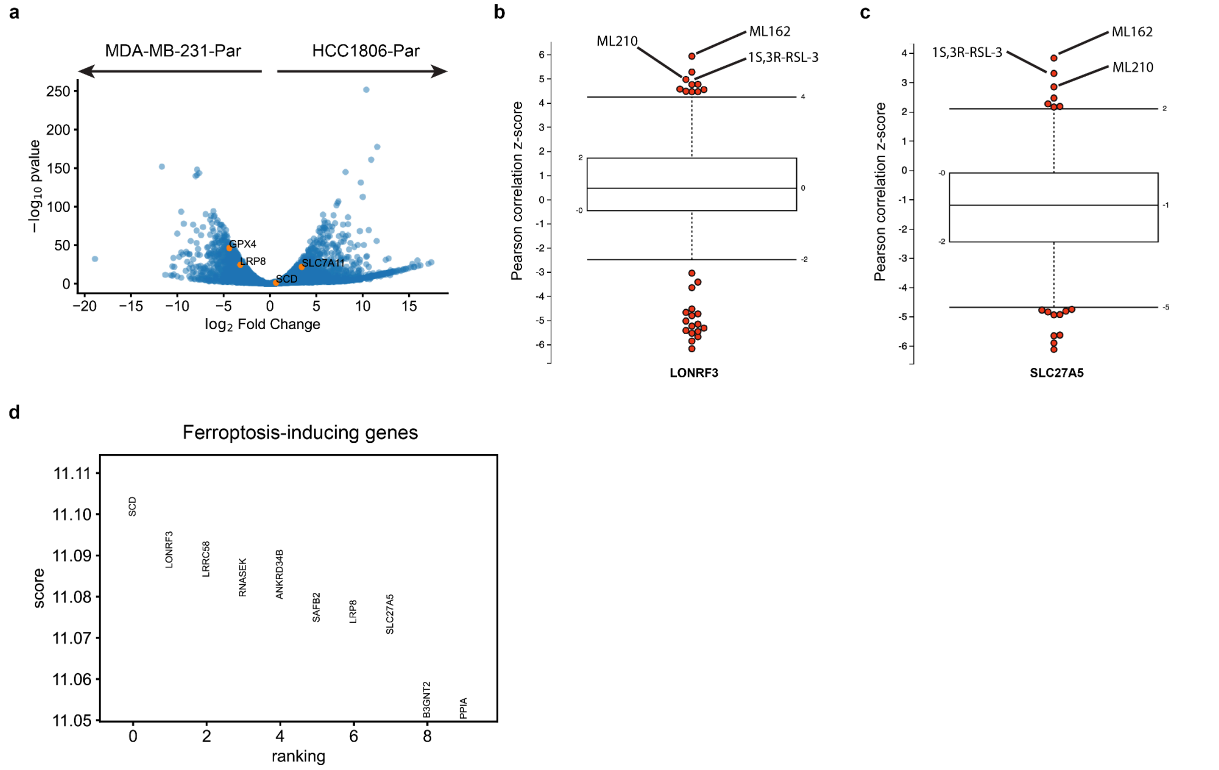

Supplementary Figure 8 | Ferroptosis analysis.

(a) DEA between MDA-MB-231-Par and HCC1806-Par with ferroptosis-associated genes in orange (n=3 independent experiments). (b) Pearson correlation z-score between highly-attributed LONRF3 expression and compound sensitivity. (c) Pearson correlation z-score between highly-attributed SLC27A5 expression and compound sensitivity. (d) Differential attribution scores of top 10 genes from ferroptosis-sensitive cell line-compound pairs relative to all others.
