# Supplementary material for: Learning chemical sensitivity reveals mechanisms of cellular response
Source: Commun Biol. 2024 Sep 15;7:1149. doi: 10.1038/s42003-024-06865-4 (PMC11402971; doi:10.1038/s42003-024-06865-4)
Supplement: Supplementary file 3 — Description of Additional Supplementary Materials [file 42003_2024_6865_MOESM3_ESM.pdf]

Description of Additional Supplementary Files

File name: Supplementary Data (tab: Supp Data 1 - Table 1)

Description: Source data for Table 1.

File name: Supplementary Data (tab Supp Data 2 - Fig1c)

Description: Source data for Figure 1c.

File name: Supplementary Data (tab: Supp Data 3 - Sup-Fig1a)

Description: Source data for Supplementary Figure 1a.

File name: Supplementary Data (tab: Supp Data 3 - Sup-Fig1c)

Description: Source data for Supplementary Figure 1c.

File name: Supplementary Data (tab: Supp Data 4 - Sup-Fig2a)

Description: Source data for Supplementary Figure 2a.

File name: Supplementary Data (tab: Supp Data 4 - Sup-Fig2b,c)

Description: Source data for Supplementary Figure 2b,c.

File name: Supplementary Data (tab: Supp Data 5 - Fig2a)

Description: Source data for Figure 2a.

File name: Supplementary Data (tab: Supp Data 5 - Fig2b)

Description: Source data for Figure 2b.

File name: Supplementary Data (tab: Supp Data 5 - Fig2c)

Description: Source data for Figure 2c.

File name: Supplementary Data (tab: Supp Data 6 - Sup-Fig3)

Description: Source data for Supplementary Figure 3.

File name: Supplementary Data (tab: Supp Data 7 - Fig3b)

Description: Source data for Figure 3b.

File name: Supplementary Data (tab: Supp Data 7 - Fig3c)

Description: Source data for Figure 3c.

File name: Supplementary Data (tab: Supp Data 7 - Fig3d)

Description: Source data for Figure 3d.

File name: Supplementary Data (tab: Supp Data 7 - Sup-Fig4-AZD7762)

Description: Source data for Supplementary Figure 4.

File name: Supplementary Data (tab: Supp Data 7 - Sup-Fig4-CAY10618)

Description: Source data for Supplementary Figure 4.

File name: Supplementary Data (tab: Supp Data 7 - Sup-Fig4-ceranib-2)

Description: Source data for Supplementary Figure 4.

File name: Supplementary Data (tab: Supp Data 7 - Sup-Fig4-ML162)

Description: Source data for Supplementary Figure 4.

File name: Supplementary Data (tab: Supp Data 8 - Fig4a-f)

Description: Source data for Figure 4a-f.

File name: Supplementary Data (tab: Supp Data 8 - Fig4g)

Description: Source data for Figure 4g.

File name: Supplementary Data (tab: Supp Data 9 - Sup-Fig5a)

Description: Source data for Supplementary Figure 5a.

File name: Supplementary Data (tab: Supp Data 9 - Sup-Fig5b)

Description: Source data for Supplementary Figure 5b.

File name: Supplementary Data (tab: Supp Data 9 - Sup-Fig5c)

Description: Source data for Supplementary Figure 5c.

File name: Supplementary Data (tab: Supp Data 10 - Sup-Fig6a)

Description: Source data for Supplementary Figure 6a.

File name: Supplementary Data (tab: Supp Data 10 - Sup-Fig6b-k)

Description: Source data for Supplementary Figure 6b-k.

File name: Supplementary Data (tab: Supp Data 11 - Fig5a,d)

Description: Source data for Figure 5a,d.

File name: Supplementary Data (tab: Supp Data 11 - Fig5b)

Description: Source data for Figure 5b.

File name: Supplementary Data (tab: Supp Data 11 - Fig5c)

Description: Source data for Figure 5c.

File name: Supplementary Data (tab: Supp Data 11 - Fig5e)

Description: Source data for Figure 5e.

File name: Supplementary Data (tab: Supp Data 11 - Fig5f;Sup-Fig7)

Description: Source data for Figure 5f and Supplementary Figure 7.

File name: Supplementary Data (tab: Supp Data 12)

Description: Module of action (ModOA) functional enrichment statistics.

File name: Supplementary Data (tab: Supp Data 13 - Fig6a)

Description: Source data for Figure 6a.

File name: Supplementary Data (tab: Supp Data 13 - Fig6b)

Description: Source data for Figure 6b.

File name: Supplementary Data (tab: Supp Data 13 - Fig6c)

Description: Source data for Figure 6c.

File name: Supplementary Data (tab: Supp Data 13 - Fig6d)

Description: Source data for Figure 6d.

File name: Supplementary Data (tab: Supp Data 13 - Fig6e)

Description: Source data for Figure 6e.

File name: Supplementary Data (tab: Supp Data 14 - Sup-Fig8a)

Description: Source data for Supplementary Figure 8a.

File name: Supplementary Data (tab: Supp Data 14 - Sup-Fig8d)

Description: Source data for Supplementary Figure 8d.
