# Supplementary material for: Learning chemical sensitivity reveals mechanisms of cellular response
Source: Commun Biol. 2024 Sep 15;7:1149. doi: 10.1038/s42003-024-06865-4 (PMC11402971; doi:10.1038/s42003-024-06865-4)
Supplement: Supplementary file 5 — Reporting Summary [file 42003_2024_6865_MOESM5_ESM.pdf]

nature portfolio

Corresponding author(s): Michael J. Keiser

Last updated by author(s): Aug 28, 2024

Reporting Summary

Nature Portfolio wishes to improve the reproducibility of the work that we publish. This form provides structure for consistency and transparency in reporting. For further information on Nature Portfolio policies, see our Editorial Policies and the Editorial Policy Checklist.

Statistics

For all statistical analyses, confirm that the following items are present in the figure legend, table legend, main text, or Methods section.

n/a	Confirmed	
☐	☒	The exact sample size (*n*) for each experimental group/condition, given as a discrete number and unit of measurement
☐	☒	A statement on whether measurements were taken from distinct samples or whether the same sample was measured repeatedly
☐	☒	The statistical test(s) used AND whether they are one- or two-sided<br>*Only common tests should be described solely by name; describe more complex techniques in the Methods section.*
☒	☐	A description of all covariates tested
☐	☒	A description of any assumptions or corrections, such as tests of normality and adjustment for multiple comparisons
☐	☒	A full description of the statistical parameters including central tendency (e.g. means) or other basic estimates (e.g. regression coefficient) AND variation (e.g. standard deviation) or associated estimates of uncertainty (e.g. confidence intervals)
☐	☒	For null hypothesis testing, the test statistic (e.g. *F*, *t*, *r*) with confidence intervals, effect sizes, degrees of freedom and *P* value noted<br>*Give P values as exact values whenever suitable.*
☒	☐	For Bayesian analysis, information on the choice of priors and Markov chain Monte Carlo settings
☒	☐	For hierarchical and complex designs, identification of the appropriate level for tests and full reporting of outcomes
☐	☒	Estimates of effect sizes (e.g. Cohen's *d*, Pearson's *r*), indicating how they were calculated

Our web collection on statistics for biologists contains articles on many of the points above.

Software and code

Policy information about availability of computer code

Data collection	Python (3.10.6) was used to collect data, along with the following packages: stringdb (0.1.5), scanpy (1.9.3).
Data analysis	Python (3.10.6) was used along with the following packages: numpy (1.23.4), pandas (1.5.1), matplotlib (3.6.2), seaborn (0.11.2), scanpy (1.9.3), scipy (1.9.3), scikit-learn (1.1.3), statsmodels (0.13.5), rdkit (2022.9.4), pytorch (1.13.0), pytorch-lightning (1.8.4). R (4.0.2) was used along with the following packages: tidyverse (1.13.0), tximport (1.18.0), genomicfeatures (1.42.2), deseq2 (1.30.1), enhancedvolcano (1.8.0), drc (3.0_1)

For manuscripts utilizing custom algorithms or software that are central to the research but not yet described in published literature, software must be made available to editors and reviewers. We strongly encourage code deposition in a community repository (e.g. GitHub). See the Nature Portfolio guidelines for submitting code & software for further information.

Data

Policy information about availability of data

All manuscripts must include a data availability statement. This statement should provide the following information, where applicable:

- Accession codes, unique identifiers, or web links for publicly available datasets
- A description of any restrictions on data availability
- For clinical datasets or third party data, please ensure that the statement adheres to our policy

Training and validation data from CTRP v1/2 can be downloaded at ftp://caftpd.nci.nih.gov/pub/OCG-DCC/CTD2/Broad/CTRPv2.0_2015_ctd2_ExpandedDataset/

CTRPv2.0_2015_ctd2_ExpandedDataset.zip. CCLE expression data can be downloaded at https://ndownloader.figshare.com/files/24613325. CCLE sample metadata can be downloaded at https://ndownloader.figshare.com/files/24613394. I-SPY2 gene expression data is located at GSE194040 (GSE194040_ISPY2ResID_AgilentGeneExp_990_FrshFrzn_meanCol_geneLevel_n988.txt.gz). I-SPY2 patient-level biomarker scores, subtype classes, and clinical/response data was gathered from supplementary information of Wolf, et. al: https://www.cell.com/cms/10.1016/j.ccell.2022.05.005/attachment/c220411b-c281-41e8-befa-a45e48af9c64/mmc3.xlsx. HGNC was used to map gene names: https://www.genenames.org/tools/multi-symbol-checker/. Protein—protein interaction data was downloaded from the STRING database v11.5. The current file version is found here: https://stringdb-downloads.org/download/protein.links.v12.0/9606.protein.links.v12.0.txt.gz. CCLE gene expression—sensitivity Pearson correlation z-scores and corresponding visualizations were obtained from the CTRP v1/2 web portal: https://portals.broadinstitute.org/ctrp.v2.1/. RNA sequencing gene expression profiles of triple negative breast cancer cell line HCC1143 WT and LRP8 KO were obtained from a data access request to Zhipeng Li as original data from related publication48. The enrichr web portal was used to perform Wikipathway, KEGG and GO enrichment analysis (https://maayanlab.cloud/Enrichr/). Source data for all tables and figures are provided with this paper.

Research involving human participants, their data, or biological material

Policy information about studies with human participants or human data. See also policy information about sex, gender (identity/presentation), and sexual orientation and race, ethnicity and racism.

Reporting on sex and gender	N/A
Reporting on race, ethnicity, or other socially relevant groupings	N/A
Population characteristics	N/A
Recruitment	N/A
Ethics oversight	N/A

Note that full information on the approval of the study protocol must also be provided in the manuscript.

Field-specific reporting

Please select the one below that is the best fit for your research. If you are not sure, read the appropriate sections before making your selection.

☒ Life sciences ☐ Behavioural & social sciences ☐ Ecological, evolutionary & environmental sciences

For a reference copy of the document with all sections, see nature.com/documents/nr-reporting-summary-flat.pdf

Life sciences study design

All studies must disclose on these points even when the disclosure is negative.

Sample size	Samples for prospective in vitro testing of model predictions were selected based on the presence of complete dose-response curves among the top 50 differential predictions for the cell line pair. The chosen predicted dose-response curves exhibited adequate Emax and Emin boundaries, appropriately covering the predicted concentration range. Given resource constraints, we reasoned that six out of 50 compounds (12%) provided adequate representativeness of model predictions.
Data exclusions	In the prospective drug screening experiment (Fig. 4a-f, Supplementary Fig. 4; Supplementary Data 7), one plate showed significant cell death in four wells within column 6. To maintain the integrity of the analysis and avoid distorting representation of the data, the values from these four wells were excluded from the analysis. Specifically, two wells were used for testing the drug CAY10618 against the cell lines HCC1806-Par and MDA-MB-231-Par. Similarly, two wells were dedicated to testing the drug neratinib against the same cell lines, HCC1806-Par and MDA-MB-231-Par.
Replication	As outlined in the methods, we initially conducted a preliminary screen to calibrate the dose-response concentration ranges of the tested compounds (300—1.7e-3 uM, 12 points) (Supplementary Fig. 4b,c; Supplementary Data 7). Based on the insights gained from the preliminary screen, we refined the concentration range for the subsequent experiment to enhance the capture of response granularity (100—2.1e-6 uM, 12 points) (Fig 4). Both experiments consistently demonstrated a significant relationship between the responses of the differential compounds (Fig. 4g, Supplementary Fig. 4c; Supplementary Data 7).
Randomization	In the context of our prospective experiments, biological sample randomization was not applicable. However, for model training and evaluation, we employed a numerical random split of samples by cell line into groups for five-fold cross-validation.
Blinding	Blinding was deemed unnecessary for our study, as the prospective experiments were solely determined by the objective predictions of an algorithm.

Reporting for specific materials, systems and methods

We require information from authors about some types of materials, experimental systems and methods used in many studies. Here, indicate whether each material, system or method listed is relevant to your study. If you are not sure if a list item applies to your research, read the appropriate section before selecting a response.

Materials & experimental systems

n/a	Involved in the study	
☒	☐	Antibodies
☐	☒	Eukaryotic cell lines
☒	☐	Palaeontology and archaeology
☒	☐	Animals and other organisms
☒	☐	Clinical data
☒	☐	Dual use research of concern
☒	☐	Plants

Methods

n/a	Involved in the study	
☒	☐	ChIP-seq
☒	☐	Flow cytometry
☒	☐	MRI-based neuroimaging

Eukaryotic cell lines

Policy information about cell lines and Sex and Gender in Research

Cell line source(s)	MDA-MB-231 (ATCC HTB-26); HCC1806 (ATCC CRL-2335)
Authentication	After receipt from ATCC, cells were authenticated using short tandem repeat (STR) analysis.
Mycoplasma contamination	All cells tested negative for mycoplasma contamination by PCR.
Commonly misidentified lines (See ICLAC register)	No commonly misidentified cell lines were used.
